# Differential Regulation of Inflammatory and Pro-Resolving Lipid Mediators in Chronic Coronary Syndrome Across Cardiometabolic Phenotypes

**DOI:** 10.3390/ijms27156667

**Published:** 2026-07-26

**Authors:** Beata Krasińska, Tomasz Urbanowicz, Katarzyna Gabriel, Maciej Kurpisz, Ewelina Grywalska, Paulina Mertowska, Ievgen Spasenenko, Giuseppe Maria Raffa, Paolo Manca, Calogera Pisano, Anna Olasińska-Wiśniewska, Krzysztof J. Filipiak, Mariusz Kowalewski, Sebastian Mertowski, Piotr Suwalski, Zbigniew Krasiński, Andrzej Tykarski

**Affiliations:** 1 Department of Hypertensiology, Angiology, and Internal Medicine, Poznań University of Medical Sciences, 61-848 Poznań, Poland; 2 Cardiac Surgery and Transplantology Department, Poznań University of Medical Sciences, 61-848 Poznań, Poland; 3 Institute of Human Genetics, Polish Academy of Sciences, 60-479 Poznań, Poland; 4 Department of Experimental Immunology, Medical University of Lublin, 20-093 Lublin, Poland; 5 Department of Research, IRCCS-ISMETT (Mediterranean Institute for Transplantation and Specialized Therapies), 90127 Palermo, Italy; 6 Department of Precision Medicine in Medical Surgical and Critical Area (Me.Pre.C.C.), University of Palermo, 90134 Palermo, Italy; 7 Department of Clinical Cardiology and Heart Failure, IRCCS ISMETT (Mediterranean Institute for Transplantation and Advanced Specialized Therapies), 90127 Palermo, Italy; 8 The Centre of Postgraduate Medical Education, 01-813 Warsaw, Poland; 9 Department of Cardiac Surgery, Center of Postgraduate Medical Education, Central Clinical Hospital of the Ministry of Interior, 02-507 Warsaw, Poland; 10 Department of Vascular, Endovascular Surgery, Angiology and Phlebology, Poznań University of Medical Sciences, 61-848 Poznań, Poland

**Keywords:** chronic coronary syndrome, resolvins, lipid mediators, inflammation, BMI, age

## Abstract

Chronic coronary syndrome (CCS) is characterized by persistent low-grade inflammation and impaired resolution of inflammatory responses within the vascular wall. Specialized pro-resolving mediators (SPMs), including resolvins and maresins, play an essential role in terminating inflammation and restoring tissue homeostasis. This prospective observational study included 83 patients hospitalized due to stable angina or angina-equivalent symptoms. Serum concentrations of leukotriene B4 (LTB4), maresin-1 (MaR1), resolvin E1 (RvE1), resolvin D1 (RvD1), and prostaglandin E2 (PGE2) were determined using enzyme-linked immunosorbent assays. Associations between mediator concentrations and clinical characteristics—including sex, age, and body mass index (BMI)—were analyzed using nonparametric statistical tests with false-discovery-rate correction. Women, patients ≥65 years of age, and individuals with BMI ≥ 30 kg/m^2^ demonstrated significantly higher concentrations of LTB4, MaR1, and RvD1. In contrast, higher RvE1 concentrations were observed in men, younger patients, and those with a BMI < 30 kg/m^2^. PGE2 levels did not differ significantly between the analyzed subgroups. Multivariable analysis identified age, LTB4, and RvD1 as independent predictors associated with coronary artery disease in the studied cohort. Circulating lipid mediator profiles differ according to sex, age, and obesity status in patients with chronic coronary syndrome. These findings demonstrate differential circulating lipid mediator profiles associated with age, sex, and obesity in patients with chronic coronary syndrome. Whether these profiles reflect adaptive inflammatory resolution or impaired resolution requires further investigation.

## 1. Introduction

Chronic low-grade inflammatory activation is a central mechanism underlying the development and progression of cardiovascular diseases, including atherosclerotic coronary artery disease, valvular pathology, and heart failure [1,2,3]. In recent years, specialized pro-resolving mediators (SPMs) have emerged as key endogenous regulators of the termination of inflammation and the restoration of tissue homeostasis [4,5,6,7]. Among these mediators, resolvins—particularly the E-series resolvins derived from eicosapentaenoic acid (EPA)—have attracted increasing attention due to their vascular and cardiometabolic relevance [8,9,10].

Unlike conventional anti-inflammatory mechanisms that mainly suppress inflammatory signaling, resolvins actively coordinate the resolution phase of inflammation. Their biological actions include regulation of cytokine release, improvement of endothelial function, enhancement of macrophage phagocytic activity, limitation of leukocyte infiltration, and modulation of lipid metabolism [11,12]. Specialized pro-resolving mediators regulate macrophage polarization toward a pro-resolving M2 phenotype.

**Figure 1:**
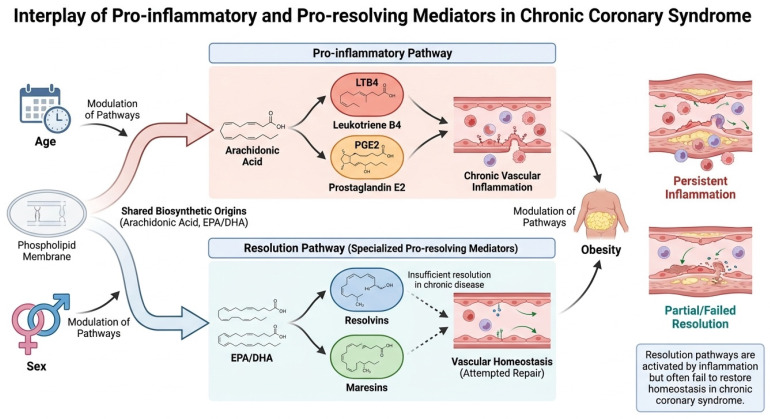
Schematic representation of the interplay between pro-inflammatory (LTB4, PGE2) and specialized pro-resolving mediators (resolvins, maresins) in chronic coronary syndrome. The diagram illustrates parallel activation of inflammatory and resolution pathways, their shared biosynthetic origins, and modulation by cardiometabolic factors such as age, sex, and obesity. The figure emphasizes that resolution pathways are activated in response to inflammation but may be insufficient to restore vascular homeostasis in chronic disease. Created with www.figurelabs.ai by Urbanowicz, T. (2026) (https://chat.figurelabs.ai/verify/FL-PUB-20260602-T001UF), accessed on 21 July 2026.

Owing to these pleiotropic effects, resolvins may represent both clinically relevant biomarkers and promising therapeutic targets in cardiovascular disorders characterized by persistent inflammatory imbalance [13,14]. Importantly, the activity of pro-resolving pathways may vary according to individual clinical and metabolic characteristics [10]. Figure 1 illustrates the dynamic interplay between pro-inflammatory and pro-resolving lipid mediators in chronic coronary syndrome, emphasizing that resolution pathways are activated in parallel with inflammatory signaling rather than as a simple inhibitory mechanism.

**Figure 2:**
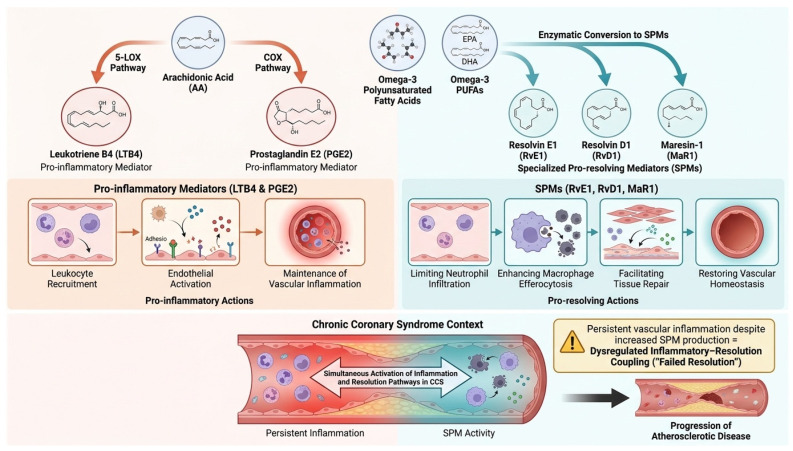
Proposed inflammatory–resolution network in chronic coronary syndrome. Schematic representation of the biosynthetic pathways and biological actions of pro-inflammatory and specialized pro-resolving lipid mediators involved in chronic coronary syndrome (CCS). Arachidonic acid is metabolized through lipoxygenase (5-LOX) and cyclooxygenase (COX) pathways to generate leukotriene B4 (LTB4) and prostaglandin E2 (PGE2), which promote leukocyte recruitment, endothelial activation, and maintenance of vascular inflammation. In parallel, omega-3 polyunsaturated fatty acids, including eicosapentaenoic acid (EPA) and docosahexaenoic acid (DHA), serve as substrates for the generation of specialized pro-resolving mediators (SPMs), such as resolvin E1 (RvE1), resolvin D1 (RvD1), and maresin-1 (MaR1). These mediators actively promote resolution of inflammation by limiting neutrophil infiltration, enhancing macrophage efferocytosis, facilitating tissue repair, and restoring vascular homeostasis. The model illustrates that chronic coronary syndrome may be characterized not by a simple excess of inflammatory mediators but by simultaneous activation of inflammatory and resolution pathways. Persistent vascular inflammation despite increased production of SPMs may reflect a state of altered inflammatory–resolution coupling or incomplete balance between inflammatory and pro-resolving pathways, contributing to the progression of atherosclerotic disease. Created with www.figurelabs.ai by Urbanowicz, T. (2026) (https://chat.figurelabs.ai/verify/FL-PUB-20260602-0BMX02), accessed on 21 July 2026.

Although inflammatory and pro-resolving lipid mediators are often presented as functionally opposing pathways, increasing evidence suggests that they are activated simultaneously during chronic inflammatory conditions. In atherosclerosis and chronic coronary syndrome, persistent inflammatory stimuli promote the generation of arachidonic acid-derived mediators such as leukotriene B4 (LTB4) and prostaglandin E2 (PGE2), which contribute to leukocyte recruitment, endothelial dysfunction, and plaque progression. In parallel, endogenous resolution pathways are activated through the biosynthesis of specialized pro-resolving mediators (SPMs), including resolvins and maresins derived from omega-3 polyunsaturated fatty acids. These mediators actively coordinate the resolution phase of inflammation by enhancing macrophage efferocytosis, limiting neutrophil infiltration, and promoting tissue repair. However, in chronic vascular disease, activation of resolution programs may be insufficient to fully counterbalance ongoing inflammatory signaling. The conceptual framework presented in Figure 2 illustrates this dynamic inflammatory–resolution network and posits that chronic coronary syndrome may be associated with distinct profiles of circulating inflammatory and pro-resolving lipid mediators. Whether these profiles represent adaptive activation of endogenous resolution pathways or impaired inflammatory resolution remains unknown.

Sex-related differences, age-associated changes in inflammatory regulation, and obesity-related disturbances in cardiometabolic homeostasis may all influence circulating levels and biological effects of resolvins and other SPMs. These factors are also strongly linked to differences in cardiovascular risk burden, lipid profile, endothelial dysfunction, and the severity of atherosclerotic disease [15,16,17,18]. Therefore, evaluating circulating pro-resolving mediators in relation to sex, age, and body mass index (BMI) may provide additional insight into the inflammatory heterogeneity of patients with chronic coronary syndrome.

In addition to quantifying individual mediators, the present study was designed to explore whether chronic coronary syndrome is characterized by coordinated remodeling of inflammatory and resolution pathways across different cardiometabolic phenotypes. We hypothesized that age, sex, and obesity influence not only inflammatory activation but also the compensatory activation of endogenous resolution programs. Accordingly, we examined circulating concentrations of LTB4, PGE2, RvD1, RvE1, and MaR1 as components of an integrated inflammatory–resolution network rather than as isolated biomarkers.

In this context, we conducted a prospective single-center study in patients hospitalized due to stable angina or angina-equivalent symptoms. The study aimed to assess the relationship between serum concentrations of selected pro-resolving mediators and the cardiometabolic profile of the analyzed population, with particular emphasis on differences associated with sex, age, and BMI.

## 2. Results

The study population included 83 patients with a median age of 71 years (63–75), of whom 35% were men (n = 29). The median body mass index was 28.4 kg/m^2^ (25.4–31.2). Among the comorbidities, hypertension was the most common [81 individuals; 98%] and dyslipidemia [77 individuals; 93%]. Diabetes was present in 31 patients (37%), chronic obstructive pulmonary disease (COPD) in 12 (15%), and coronary artery disease confirmed by coronary angiography was diagnosed in 34 patients (41%).

Laboratory tests showed a median leukocyte count of 7.29 × 10^9^/L (5.94–8.87), hemoglobin concentration of 8.55 mmol/L (7.68–9.15), and platelet count of 212 × 10^9^/L (177–266). Median alanine aminotransferase (ALT) activity was 24 U/L (19–37), and creatinine concentration was 0.89 mg/dL (0.73–1.12). The median total cholesterol level was 137 mg/dL (133–166), high-density lipoprotein (HDL) 50 mg/dL (40–60), low-density lipoprotein (LDL) 76 mg/dL (58–103), and triglycerides 105 mg/dL (75–128). Median fasting glucose was 99 mg/dL (90–117), and glycemic hemoglobin (HbA1c) was 6.2% (5.7–7.2). Median uric acid level was 5.20 mg/dL (4.38–6.24), and Creatine Kinase–Myocardial Band (CK-MB) was 3.30 (1.50–7.40).

The most commonly used pharmacological treatments included aspirin [83 patients; 100%], statins [83; 100%], beta-blockers [67; 75%], and angiotensin-converting enzyme (ACE) inhibitors [58; 60%]. Loop diuretics were used by 39 patients (44%), sodium-glucose co-transporter 2 (SGLT2) inhibitors by 35 (39%), mineralocorticoid receptor antagonists (MRA) by 25 (28%), and angiotensin receptor blockers (ARBs) by 15 patients (17%).

On coronary angiograms, epicardial coronary artery disease was observed in 34 patients and located in the left descending artery (*n* = 19), circumflex artery (n = 17), and right coronary artery (n = 13). A total of 24 (27% of the analyzed population) patients required percutaneous interventions.

The lipid mediators obtained from the peripheral blood samples were analyzed, including serum concentration of leukotriene B4 (LTB4) (168.6 (70.0–417.1) pg/mL), maresin 1 (MaR1) (21.89 (10.64–54.73) ng/mL), resolvin E1 (RvE1) (5.91 (5.15–8.18) ng/mL), resolvin D1 (RvD1) (389.7 (211.0–707.0) pg/mL), and prostaglandin E2 (PGE2) (201.5 (165.5–241.7) pg/mL).

To better characterize the relationship between inflammatory activation and resolution pathways, Spearman correlation analyses were performed among all lipid mediators. Significant positive correlations were observed between LTB4 and MaR1 (r = 0.761, *p* < 0.001), LTB4 and RvD1 (r = 0.762, *p* < 0.001), and between MaR1 and RvD1 (r = 0.749, *p* < 0.001), whereas no significant correlations were found for PGE2 or RvE1 (Table 1).

Notably, RvE1 did not correlate significantly with either LTB4, MaR1, or RvD1, suggesting that E-series resolvins may be regulated independently from D-series resolvins and maresins in patients with chronic coronary syndrome.

### 2.1. Sex-Related Differences in Clinical Profile and Lipid Mediators

The study group included 29 (35%) men and 54 (65%) women. Women had significantly lower body weight and shorter height than men. Median body weight was 73.00 kg (69.00–84.00) in women and 83.50 kg (77.00–93.00) kg in men, respectively (*p* = 0.006), while median height was 160.00 cm (156.00–166.00) versus 173.50 cm (170.00–176.00) (*p* < 0.001). No significant differences were found in BMI, although a trend toward higher BMI values was observed in women: 30.48 (25.97–32.00) kg/m^2^ vs. 28.37 (25.35–30.42) kg/m^2^; *p* = 0.080.

No significant sex differences were observed in the prevalence of hypertension, diabetes, stroke, peripheral artery disease (PAD), smoking, or chronic obstructive pulmonary disease (COPD) (Table 2). Regarding angiographic parameters, no significant differences were observed between women and men in the prevalence of atherosclerotic involvement of the left descending artery (LAD), circumflex artery (Cx), or right coronary artery (RCA). There were also no significant differences in most of the laboratory parameters assessed, including peripheral blood counts, inflammatory markers, glucose levels, renal parameters, and lipid profile. Only total cholesterol showed a trend towards higher values in women: 141.00 mg/dL (130.00–173.00) vs. 129.50 mg/dL (116.00–165.00); *p* = 0.064 (Table 1).

The most pronounced differences between women and men were found in the concentrations of the lipid mediators studied. Women had significantly higher concentrations of LTB4, MaR1, and RvD1. The median LTB4 concentration was 320.37 pg/mL (157.50–654.41) in women and 128.70 pg/mL (40.18–216.72) in men (*p* < 0.001). For MaR1, these values were 54.66 ng/mL (26.56–69.56) and 15.67 ng/mL (6.52–29.32), respectively (*p* < 0.001), while for RvD1 646.70 pg/mL (434.30–798.89) vs. 245.45 pg/mL (145.50–611.34) (*p* < 0.001). A different relationship was observed for RvE1, with higher concentrations in men: 6.43 ng/mL (5.64–9.21) vs. 5.49 ng/mL (4.72–5.75) in women (*p* < 0.001). No significant difference in PGE2 concentration was found between sexes (*p* = 0.133) Detailed lipid profile parameters, including total cholesterol, HDL, and LDL levels, are presented in Table 3.

#### Multivariable Model Associated with Angiographically Confirmed Coronary Artery Disease + ROC

The multivariable analysis included the following parameters: age, comorbidities (arterial hypertension, dyslipidemia, diabetes mellitus), smoking, obesity (BMI > 30 kg/m^2^), HDL/LDL ratio, and lipid markers (LTB4, MaR1, RvD1, RvE1, PGE2). A multivariable logistic regression model was constructed to identify factors associated with angiographically confirmed coronary artery disease within the study cohort.

Because circulating lipid mediator concentrations are expressed in picograms per milliliter or nanograms per milliliter, the corresponding odds ratios are calculated for a one-unit increase in concentration. Consequently, odds ratios close to unity should not be interpreted as indicating negligible biological effects but rather reflect the small measurement increment used in the regression model. Larger concentration differences commonly observed between individuals correspond to proportionally greater cumulative changes in estimated risk. Therefore, the reported odds ratios primarily describe the direction and statistical significance of the associations rather than their magnitude per clinically meaningful concentration change.

The multivariable model indicated the predictive value of 3 components: age (OR: 1.18, 95% CI: 1.03–1.35, *p* = 0.021), LTB4 (OR: 0.99, 95% CI: 0.98–0.99, *p* = 0.002) (per 1 pg/mL increase), and RvD1 (OR: 1.01, 95% CI: 1.00–1.02, *p* = 0.001) (per 1 pg/mL increase). The reported odds ratios correspond to a one-unit increase in the original concentration units (pg/mL or ng/mL), consistent with the regression model’s analytical output.

**Figure 3:**
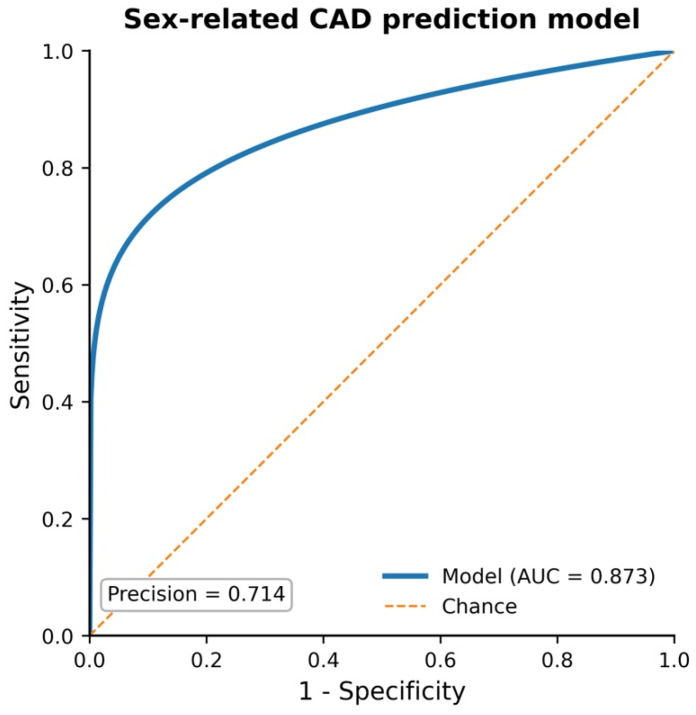
Receiver Operating Characteristic Curve for the Multivariable CAD Association Model.

The receiver operating characteristic curve (ROC) showed an area under the curve of 0.873 and a precision of 0.714, as shown in Figure 3.

### 2.2. BMI-Related Differences in Clinical Profile and Lipid Mediators

After dividing the study population by body mass index, no differences in age or height were found between patients with a BMI < 30 kg/m^2^ and those with a BMI ≥ 30 kg/m^2^. According to the adopted criterion, individuals with a BMI ≥ 30 kg/m^2^ had significantly higher body weight than those with a BMI < 30 kg/m^2^ (91.00 [83.00–95.00] kg vs. 76.00 [67.00–82.00] kg, *p* < 0.001) (Table 4).

Regarding metabolic parameters, the group with BMI ≥ 30 kg/m^2^ had higher total and HDL cholesterol levels. The median total cholesterol level was 142.00 (129.00–183.00) mg/dL in patients with BMI ≥ 30 and 129.00 (116.00–155.00) mg/dL in patients with BMI < 30 kg/m^2^ (*p* = 0.016). Similarly, HDL levels were higher in the obese group: 56.50 (46.00–64.00) mg/dL vs. 46.00 (39.00–57.00) mg/dL (*p* = 0.010). Other laboratory parameters, including glucose, HbA1c, creatinine, LDL, and triglycerides, did not differ significantly between the groups (Table 5).

The most pronounced differences between groups were observed in lipid mediator concentrations. Patients with a BMI ≥ 30 kg/m^2^ had higher concentrations of LTB4, MaR1, and RvD1. The median LTB4 concentration was 303.39 (122.33–654.41) pg/mL in the BMI ≥30 group versus 132.08 (40.17–211.37) pg/mL in the BMI < 30 kg/m^2^ group (*p* = 0.002). For MaR1, these values were 54.66 (23.38–74.91) ng/mL and 15.67 (6.83–25.06) ng/mL, respectively (*p* < 0.001), while for RvD1, 650.54 (391.24–798.89) pg/mL versus 242.64 (145.50–408.16) pg/mL (*p* < 0.001). An inverse relationship was observed for RvE1, with higher concentrations in patients with BMI < 30 kg/m^2^: 6.60 (5.72–9.36) ng/mL vs. 5.48 (4.74–5.89) ng/mL (*p* < 0.001). However, no differences were found in PGE2 concentration (*p* = 0.528) (as presented in Table 5).

#### Multivariable Model Associated with Angiographically Confirmed Coronary Artery Disease in Obese Patients + ROC

The multivariable analysis included the following parameters: age, comorbidities (arterial hypertension, dyslipidemia, diabetes mellitus), smoking, HDL/LDL ratio, and lipid markers (LTB4, MaR1, RvD1, RvE1, PGE2).

**Figure 4:**
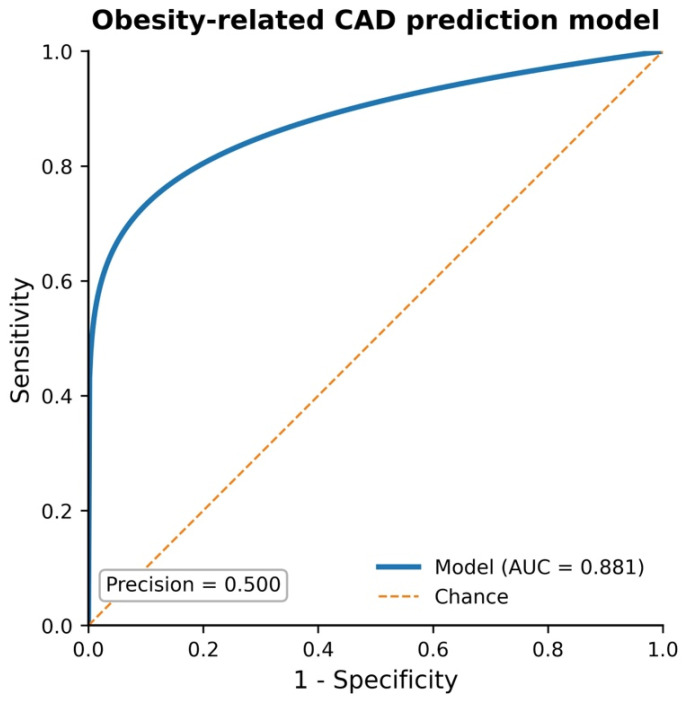
Receiver Operating Characteristic Curve for the Obesity-Stratified CAD Association Model.

After adjustment for potential confounders, the multivariable analysis for obesity-related CAD prediction, including age (OR: 1.35, 95% CI: 1.03–1.78, *p* = 0.031) and LDL/HDL ratio (OR: 6.55, 95% CI: 1.52–28.11, *p* = 0.012), was significant. The receiver operating characteristic curve for the presented model showed an area under the curve (AUC) of 0.881 and a precision of 0.500, as shown in Figure 4.

### 2.3. Age-Related Differences in Clinical, Angiographic, and Lipid Mediator Profiles

The age distribution of the study population showed significant differences, primarily in selected biochemical parameters and lipid mediator concentrations. As expected, the median age was significantly lower in the < 65-year-old group than in the ≥ 65-year-old group (59.50 [55.50–62.50] vs. 73.00 [70.00–77.00] years, *p* < 0.001) (Table 6).

Compared with younger patients, patients aged ≥65 years had higher total and HDL cholesterol levels. The median total cholesterol level was 141.00 (125.00–180.00) mg/dL in older patients and 123.00 (109.00–133.00) mg/dL in patients <65 years of age (*p* < 0.001). Similarly, HDL levels were higher in the older group: 56.00 (46.00–63.00) mg/dL vs. 41.50 (36.50–46.00) mg/dL (*p* < 0.001). Patients aged 65 years and older had higher MCHC values (21.10 [20.65–21.30] mmol/L vs. 20.70 [20.30–21.00] mmol/L, *p* = 0.011) and higher creatinine concentrations (0.96 [0.81–1.17] mg/dL vs. 0.84 [0.69–1.12] mg/dL, *p* = 0.027). Regarding clinical characteristics, younger patients were more likely to have higher body weight than older individuals (88.50 [79.00–97.00] kg vs. 80.00 [70.00–88.00] kg, *p* = 0.010). However, no significant differences were found in height, BMI, prevalence of hypertension, diabetes, PAD, or COPD. The most pronounced differences between age groups were related to lipid mediators. In patients aged ≥65, significantly higher concentrations of LTB4, MaR1, and RvD1 were found. The median LTB4 concentration was 217.95 (94.57–532.86) pg/mL in the older group and 92.65 (26.54–193.36) pg/mL in the younger group (*p* = 0.007). For MaR1, these values were 34.73 (14.75–64.62) ng/mL and 11.05 (4.73–21.26) ng/mL, respectively (*p* < 0.001), while for RvD1 it was 475.80 (260.82–780.45) pg/mL vs. 221.37 (123.99–363.32) pg/mL (*p* = 0.011). An inverse relationship was observed for RvE1, with higher concentrations in patients <65 years of age: 8.59 (5.95–11.03) ng/mL vs. 5.69 (4.90–6.50) ng/mL (*p* < 0.001). PGE2 concentration did not differ significantly between age groups (*p* = 0.694) (Table 7).

#### Multivariable Analysis of Age-Related CAD Prediction + ROC

The multivariable analysis included the following parameters: sex, comorbidities (arterial hypertension, dyslipidemia, diabetes mellitus), smoking, obesity (BMI > 30 kg/m^2^), HDL/LDL ratio, and lipid markers (LTB4, MaR1, RvD1, RvE1, PGE2).

After adjustment for potential confounders, the multivariable model indicated that circulating LTB4 was predictive of CAD diagnosis (OR: 0.99, 95% CI: 0.98–0.99, *p* = 0.049) (per 1 pg/mL increase) among patients aged 65 years or older.

**Figure 5:**
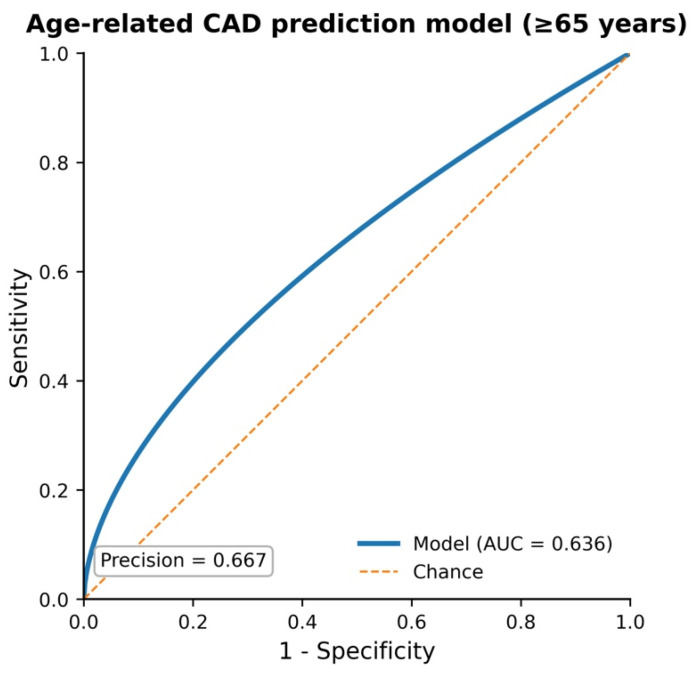
Receiver Operating Characteristic Curve for the Age-Related CAD Prediction Model (≥65 Years).

The receiver operating characteristic curve (ROC) showed an area under the curve of 0.636 and a precision of 0.667 (Figure 5).

## 3. Discussion

Our findings indicate simultaneous activation of inflammatory and pro-resolving lipid mediators. Although this pattern is consistent with altered inflammatory–resolution biology, the present study cannot determine whether these responses represent effective compensation or defective resolution, as no healthy comparator or functional assessment of resolution pathways was included.

Therefore, our findings support the concept that CCS may represent an altered inflammatory–resolution coupling, with coordinated alterations in both inflammatory and pro-resolving lipid mediator pathways across distinct cardiometabolic phenotypes. Rather than observing an isolated increase in inflammatory mediators, we identified simultaneous elevations of LTB4, MaR1, and RvD1 in older, obese, and female patients. This pattern suggests that persistent inflammatory activation is accompanied by compensatory activation of endogenous resolution mechanisms. Therefore, our findings support the concept that CCS may represent a state of altered inflammatory–resolution coupling, characterized by activation of both pathways but insufficient restoration of vascular homeostasis.

An alternative interpretation should also be considered. Simultaneous elevation of pro-inflammatory mediators together with specialized pro-resolving mediators may reflect a physiological compensatory response to persistent vascular inflammation rather than defective resolution itself. Because specialized pro-resolving mediators are actively synthesized during ongoing inflammation, increased circulating concentrations may indicate activation of endogenous homeostatic mechanisms attempting to restore tissue balance. Distinguishing adaptive activation from impaired resolution would require functional assays, longitudinal sampling, or comparison with healthy individuals, none of which were available in the present study.

The biosynthetic origin of the analyzed mediators provides a plausible mechanistic framework for these observations. LTB4 is generated predominantly through 5-lipoxygenase-mediated metabolism of arachidonic acid in neutrophils, monocytes, macrophages, and vascular inflammatory cells. PGE2 originates from cyclooxygenase-dependent metabolism of arachidonic acid and is produced by endothelial cells, vascular smooth muscle cells, platelets, and infiltrating leukocytes. In contrast, RvD1 and MaR1 derive from docosahexaenoic acid, whereas RvE1 is generated from eicosapentaenoic acid through coordinated lipoxygenase-mediated pathways. Consequently, circulating concentrations of these mediators likely represent an integrated systemic signal originating from both vascular lesions and circulating immune cells rather than direct measurements of plaque-level activity.

Circulating concentrations of the analyzed mediators likely reflect contributions from multiple cellular compartments involved in atherosclerosis, including neutrophils, monocytes/macrophages, endothelial cells, vascular smooth muscle cells, and platelets. Consequently, serum measurements should be interpreted as integrated systemic signals rather than direct indicators of plaque-specific mediator production.

Specialized pro-resolving mediators derived from omega-3 fatty acids regulate macrophage polarization, endothelial activation, and leukocyte trafficking. Therefore, disturbances in the balance between LTB4 and resolvin pathways may reflect impaired resolution of vascular inflammation in chronic coronary syndrome. It should be emphasized that the calculated odds ratios reflect associations within the studied CCS population rather than comparisons with a non-diseased control group. Moreover, the reported estimates correspond to a one-unit increase in the original assay units (pg/mL or ng/mL). Because these units represent very small concentration increments, odds ratios are expected to remain numerically close to 1.0 even when statistically significant associations are present. Therefore, these estimates should be interpreted primarily as indicators of the direction and strength of the association within the study cohort rather than as measures of clinically meaningful risk associated with larger concentration differences or as estimates applicable to the general population.

The correlation analysis provided additional insight into the relationship between pro-inflammatory and pro-resolving lipid mediator pathways. We observed strong positive correlations between LTB4 and the specialized pro-resolving mediators RvD1 (Spearman r = 0.762, *p* < 0.001) and MaR1 (Spearman r = 0.761, *p* < 0.001). These findings suggest that increased inflammatory activation in chronic coronary syndrome is accompanied by a parallel activation of endogenous resolution mechanisms. Rather than reflecting a purely suppressive response, specialized pro-resolving mediators are biosynthesized in response to inflammatory stimuli and actively orchestrate the resolution phase by limiting neutrophil infiltration, enhancing macrophage efferocytosis, and promoting tissue repair.

Classical inflammatory models predict reciprocal regulation of inflammatory and anti-inflammatory pathways. However, the strong positive correlations observed between LTB4, MaR1, and RvD1 challenge this simplistic paradigm. Instead, our data support an alternative model in which chronic inflammatory stimulation simultaneously induces endogenous resolution pathways. Although increased circulating SPM concentrations were observed alongside elevated inflammatory mediators, the present study cannot determine whether resolution programs are functionally insufficient, as no functional assessment of inflammatory resolution was performed. This phenomenon resembles the concept of incomplete balance between inflammatory and pro-resolving pathways described in chronic inflammatory diseases and may represent an underrecognized mechanism contributing to atherosclerotic persistence.

In the context of our findings, the parallel increase in both LTB4 and pro-resolving mediators may reflect a state of persistent inflammatory activation accompanied by compensatory resolution signaling [19,20]. This pattern is compatible with persistent inflammatory activation accompanied by endogenous activation of specialized pro-resolving mediators.

The strong correlation observed between MaR1 and RvD1 (Spearman r = 0.749, *p* < 0.001) further supports the concept of coordinated regulation of pro-resolving mediator networks. Both mediators are derived from docosahexaenoic acid through lipoxygenase-dependent pathways and share partially overlapping biological functions, including modulation of macrophage polarization and restoration of endothelial homeostasis. Taken together, these observations are consistent with the concept of a dynamic inflammatory–resolution balance in atherosclerosis, in which persistent vascular inflammation triggers compensatory activation of specialized pro-resolving pathways that may represent an adaptive attempt to restore immune and vascular homeostasis.

Specialized pro-resolving mediators are biosynthesized from omega-3 polyunsaturated fatty acids via lipoxygenase-dependent pathways and exert their effects via specific receptors, such as ALX/FPR2 and ChemR23 [21]. These mediators actively promote the termination of inflammation by enhancing macrophage efferocytosis, limiting neutrophil infiltration, and restoring endothelial function [22]. Dysregulation of these pathways has been implicated in the persistence of vascular inflammation and progression of atherosclerotic disease. The measured lipid mediators originate from multiple biological sources, including activated leukocytes, endothelial cells, and vascular smooth muscle cells [23]. Their biosynthesis involves enzymatic pathways such as cyclooxygenases (COX), lipoxygenases (LOX), and cytochrome P450 enzymes [10,24]. Leukotriene B4 is primarily generated via the 5-lipoxygenase pathway, whereas resolvins and maresins are derived from omega-3 fatty acids through sequential lipoxygenase-mediated reactions [25]. These pathways are tightly regulated and influenced by systemic metabolic and inflammatory conditions.

The most consistent differences were observed for LTB4, MaR1, RvE1, and RvD1. Women, patients aged ≥65 years, and those with a BMI ≥ 30 kg/m^2^ had higher concentrations of LTB4, MaR1, and RvD1, whereas higher RvE1 concentrations were observed in men, younger individuals, and patients with a BMI < 30 kg/m^2^. These results suggest that individual lipid mediators may reflect distinct elements of the inflammatory response and its resolution processes, and their profiles are closely linked to the patient’s clinical and metabolic phenotype.

When interpreting these observations, it is important to emphasize that chronic coronary artery disease develops in conditions of coexisting persistent inflammatory activation, endothelial dysfunction, and metabolic abnormalities [26,27,28]. Under such conditions, one can expect both increased synthesis of proinflammatory mediators and compensatory activation of pro-resolving pathways. Higher LTB4 concentrations in selected study subgroups may reflect greater inflammatory activation, while increased MaR1 and RvD1 concentrations may reflect secondary activation of mechanisms responsible for resolving inflammation. This pattern seems particularly likely in older patients and those with a higher BMI, in whom chronic inflammation and metabolic abnormalities are typically more severe.

The divergent behavior of RvE1 compared with RvD1 and MaR1 suggests that E-series and D-series resolution pathways may be differentially regulated in chronic coronary syndrome. Since RvE1 is generated from EPA whereas RvD1 and MaR1 derive from DHA, the observed differences may reflect distinct substrate availability, enzymatic regulation, receptor expression, or mediator turnover. This finding raises the possibility that reduced RvE1 concentrations may represent an early marker of impaired resolution capacity despite compensatory activation of other specialized pro-resolving pathways.

**Figure 6:**
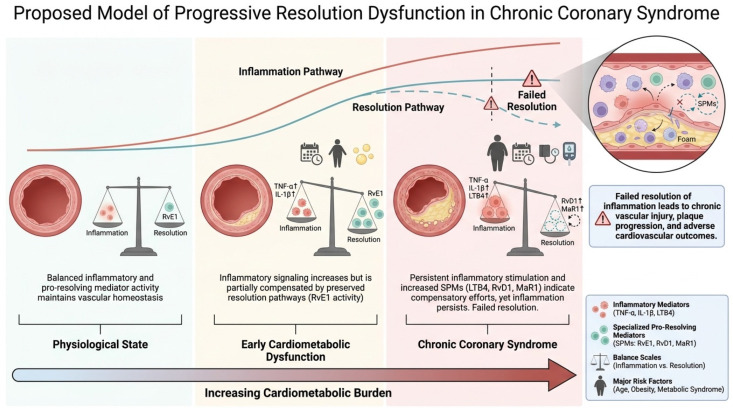
Proposed model of progressive resolution dysfunction in chronic coronary syndrome. The schematic illustrates a hypothetical evolution of inflammatory and specialized pro-resolving mediator responses across increasing cardiometabolic burden. Under physiological conditions, inflammatory activation is balanced by effective resolution mechanisms, maintaining vascular homeostasis. In the early stages of cardiometabolic dysfunction, inflammatory signaling increases but remains partially compensated by preserved resolution pathways, including RvE1 activity. With advancing age, obesity, and accumulation of cardiovascular risk factors, persistent inflammatory stimulation is accompanied by increased concentrations of LTB4, RvD1, and MaR1, suggesting activation of endogenous compensatory resolution programs. Despite this response, vascular inflammation persists, indicating that specialized pro-resolving mediator activity becomes insufficient to fully restore tissue homeostasis. This state of altered inflammatory–resolution coupling, referred to as incomplete balance between inflammatory and pro-resolving pathways, may contribute to chronic vascular injury, plaque progression, and adverse cardiovascular outcomes in chronic coronary syndrome. Created in www.figurelabs.ai by Urbanowicz, T. (2026) (https://chat.figurelabs.ai/verify/FL-PUB-20260602-N5BOU9), accessed on 21 July 2026.

Based on the observed mediator profiles and correlation structure, we propose a conceptual model describing the evolution of inflammatory–resolution interactions in chronic coronary syndrome. This framework integrates the parallel increases in LTB4, RvD1, and MaR1, together with the decline in RvE1 observed in older, obese, and female patients, and provides a biological explanation for the persistence of vascular inflammation despite activation of endogenous resolution pathways (Figure 6).

The results regarding RvE1 are particularly interesting, as this mediator showed a direction of change opposite to LTB4, MaR1, and RvD1. Higher RvE1 concentrations in men, younger patients, and those with a BMI < 30 kg/m^2^ may suggest that E-series resolvin pathway activity remains better preserved in patients with a relatively lower metabolic burden. This may indicate that, in a more advanced cardiometabolic phenotype, an imbalance between the various axes of inflammation regulation and resolution occurs and that RvE1 reflects a different aspect of the biological response than RvD1 or MaR1.

Unlike RvD1 and MaR1, which were elevated in older and obese individuals, RvE1 demonstrated an opposite pattern. This divergence suggests that E-series and D-series resolution pathways may be differentially regulated during chronic vascular inflammation. Because RvE1 derives from EPA whereas RvD1 and MaR1 derive from DHA, the observed differences may reflect distinct substrate availability, enzymatic regulation, receptor expression, or metabolic turnover. An alternative explanation is that declining RvE1 concentrations represent an early marker of impaired resolution capacity, whereas elevated RvD1 and MaR1 reflect compensatory activation secondary to persistent inflammatory stimulation. Further lipidomic studies are required to determine whether these pathways play complementary or divergent roles in atherosclerotic disease progression.

Our results also suggest that the relationship between mediators’ profiles and lipid metabolism is significant [29]. In both age and BMI analyses, higher total cholesterol and HDL levels were observed in older patients and those with a BMI ≥ 30 kg/m^2^, respectively. This is significant because lipid abnormalities remain closely associated with endothelial dysfunction, oxidative stress, and the progression of atherosclerosis. In particular, the role of HDL is not limited solely to reverse cholesterol transport but also encompasses modulation of endothelial function, the inflammatory response, and oxidative stress [30]. It can therefore be assumed that the observed differences in lipid mediator concentrations partly reflect complex interactions between chronic inflammation and lipid metabolism.

It is also noteworthy that in our study, PGE2 levels did not differ significantly between the analyzed subgroups. However, this does not rule out its involvement in the pathophysiology of chronic coronary artery disease. PGE2 is a mediator with broad-spectrum immunomodulatory effects and may influence cytokine production, inflammatory cell activity, and local tissue response [31]. Its synthesis depends, among other things, on the activity of COX-2 and mPGES-1, and available data indicate that this pathway may be modulated by an environment rich in cholesterol and LDL, which can enhance macrophage inflammatory response [32]. Therefore, the lack of differences between groups in our study does not necessarily imply a lack of biological significance of PGE2 but may instead indicate that its role is more dependent on local tissue conditions than on sex, age, or BMI.

Sex hormones may contribute to the observed differences in mediator profiles. Experimental studies have demonstrated that estrogen influences both arachidonic acid metabolism and specialized pro-resolving mediator biosynthesis, enhancing the activity of several resolution pathways. Consequently, the higher concentrations of MaR1 and RvD1 observed in women may reflect sex-specific regulation of inflammatory adaptation rather than simply greater inflammatory burden. This possibility is consistent with increasing recognition of sex-dependent immune regulation in cardiovascular disease.

It should also be acknowledged that the biological relevance and quantification of circulating specialized pro-resolving mediators remain a matter of ongoing debate, and recent studies have questioned both their detectability and functional significance in human plasma. A previous report [33] challenged SPMs’ role as endogenous mediators of the inflammation resolution. Circulating oxylipin levels may not reflect local tissue production, and potential influencing factors, such as metabolism, transport, and degradation, are highlighted [34]. The potential challenges in applying standard limit-of-detection or limit-of-quantitation criteria to specialized pro-resolving mediator analysis need to be addressed [35].

It is also worth relating our results to pharmacotherapy, particularly statin therapy. In the study population, no clear relationship was observed, suggesting a simple, dose-dependent effect of statin therapy intensity on the concentrations of the analyzed mediators. This is somewhat inconsistent with previous reports indicating that high-intensity statins may exhibit anti-inflammatory and antithrombotic effects, including by influencing neutrophil extracellular trap (NET) formation [36,37]. Our observations suggest that the effect of statins on pro-resolving pathways and lipid mediators is more complex, dependent on the patient’s phenotype, disease progression, and concomitant metabolic disorders, rather than solely on the intensity of lipid-lowering treatment.

In parallel with lipid mediator pathways, attention has increasingly focused on systemic inflammatory biomarkers derived from routine hematological parameters. Ratios reflecting leukocyte subpopulations, such as the monocyte-to-lymphocyte ratio (MLR), together with erythrocyte indices, such as mean corpuscular hemoglobin concentration (MCHC), have been proposed as indicators of vascular inflammatory activity and atherosclerotic burden [38,39]. Previous investigations have suggested that these parameters may serve as predictors of collateral carotid artery disease, highlighting the importance of immune–vascular interactions in the development of advanced atherosclerosis [40,41].

The higher proportion of angiographically confirmed coronary lesions observed in men may reflect referral bias or differences in clinical presentation rather than true prevalence differences. Given the absence of a control group, the calculated odds ratios should be interpreted strictly as measures of association within the CCS cohort rather than as indicators of disease risk or diagnostic performance.

An alternative interpretation of the present findings is that the simultaneous increase in pro-inflammatory and specialized pro-resolving mediators represents an adaptive compensatory response rather than impaired resolution itself. Because only circulating concentrations were measured, the present study cannot distinguish between effective activation of endogenous resolution programs and insufficient biological activity at the tissue level. Future mechanistic studies integrating functional assays and tissue-specific analyses will be necessary to clarify whether these mediator profiles reflect successful compensation or biologically ineffective resolution.

Although the present study primarily focused on lipid mediators regulating the resolution phase of inflammation, the observed associations between cardiometabolic phenotype and mediator concentrations are consistent with the broader concept that systemic inflammatory markers contribute to the characterization of vascular disease. In this regard, previously reported relationships between MLR, MCHC, and carotid collateral circulation provide complementary evidence supporting the relevance of inflammatory biomarkers in cardiovascular risk stratification and reinforce the biological significance of inflammatory imbalance in atherosclerotic disease.

Interpretation of circulating lipid mediator concentrations should also consider potential effects of concomitant pharmacological treatment, dietary omega-3 intake, metabolic status, and other systemic inflammatory conditions. These variables may influence the biosynthesis of specialized pro-resolving mediators independently of coronary artery disease and could contribute to the interindividual variability observed in the present cohort.

The angiographic results are also interesting. Patients with a BMI < 30 kg/m^2^ had more coronary artery involvement, while those in the BMI ≥ 30 kg/m^2^ group were more likely to have no significant coronary artery disease. However, in the ≥ 65-year-old group, LAD involvement was more common. Although these observations should be interpreted with caution, they may indicate that the relationship between the inflammatory–proresolving profile and the angiographic appearance is not linear and is modulated by age, sex, obesity, and chronic treatment.

From a clinical perspective, these findings suggest that circulating lipid mediator profiles may provide insight into the inflammatory heterogeneity of patients with chronic coronary syndrome. Although the present results do not support immediate clinical application, characterization of inflammatory–resolution networks may contribute to future biomarker-guided cardiovascular risk stratification and facilitate development of therapies targeting resolution pathways rather than inflammation alone.

The present findings also define several priorities for future translational research. The first objective should be longitudinal characterization of inflammatory–resolution mediator profiles during the progression of chronic coronary syndrome to determine whether distinct lipid mediator signatures precede clinical deterioration or reflect compensatory adaptation. The second priority is to identify interventions that restore physiological inflammatory–resolution coupling rather than simply suppress inflammation. Such approaches may include dietary omega-3 enrichment, selective stimulation of endogenous specialized pro-resolving mediator biosynthesis, development of stable SPM analogs, or modulation of receptors involved in resolution signaling. Ultimately, combining lipid mediator profiling with clinical risk assessment may enable identification of patients most likely to benefit from personalized resolution-directed therapeutic strategies.

A better understanding of inflammatory resolution will require integration of circulating lipid mediator measurements with functional studies of immune-cell behavior, receptor expression, intracellular signaling, and tissue-specific inflammatory responses. Future investigations combining lipidomics, transcriptomics, and single-cell technologies may clarify whether elevated circulating specialized pro-resolving mediators represent effective activation of endogenous resolution programs, compensatory but insufficient responses, or impaired downstream signaling despite preserved mediator production. Such mechanistic studies are essential before resolution-directed therapies can be translated into clinical practice.

Taken together, the present findings should be interpreted within the framework of systemic inflammatory–resolution coupling rather than as evidence of direct causal involvement of individual mediators in disease pathogenesis. The observed parallel increase in both pro-inflammatory and pro-resolving lipid mediators is consistent with the concept that chronic vascular inflammation is accompanied by compensatory activation of endogenous resolution pathways. Importantly, the variability of these responses across sex, age, and obesity strata suggests that cardiometabolic phenotype modulates not only inflammatory burden but also the capacity for resolution. In this context, circulating mediator profiles may reflect an integrated systemic response rather than isolated pathway activation, highlighting the need for future studies.

Strengths of the present study include simultaneous assessment of both inflammatory and specialized pro-resolving lipid mediators, analysis across multiple cardiometabolic phenotypes, and evaluation of mediator network interactions rather than isolated biomarkers. Finally, these findings support the concept that chronic coronary syndrome is characterized by altered inflammatory–resolution coupling rather than isolated pathway activation.

### Study Limitations

The present study has several important limitations. First, the relatively small sample size and single-center design limit the generalizability of the findings. Second, a key limitation of the present study is the absence of a healthy control group. Therefore, the findings should not be interpreted as disease-specific alterations but rather as associations within a population of patients with chronic coronary syndrome. Moreover, inclusion of age-matched individuals without angiographically confirmed coronary artery disease would allow discrimination between physiological aging-associated changes in lipid mediator homeostasis and disease-specific inflammatory–resolution dysregulation. Consequently, the observed relationships reflect intra-cohort variability and cannot establish whether the measured lipid mediator profiles differ from physiological conditions. Future studies including matched healthy controls are required to determine the pathophysiological specificity of these findings.

Third, the observational design does not allow causal inference, and the reported associations should be considered hypothesis-generating. Fourth, lipid mediator quantification was performed using ELISA-based assays, which may be subject to limited specificity and potential overestimation compared with LC–MS/MS-based lipidomic approaches. Recent studies [42,43] have raised concerns about the reliability of ELISA-based quantification of specialized pro-resolving mediators, with potential overestimation and limited specificity compared with LC–MS/MS-based lipidomic approaches. Additionally, ELISA enables standardized quantification of circulating lipid mediators; LC-MS/MS remains the analytical reference standard because of its higher specificity and ability to distinguish structurally related lipid mediators. As mentioned before, the multivariable regression models should be interpreted as exploratory because the relatively small number of angiographically confirmed coronary artery disease (only 34 patients) events may increase the risk of overfitting. These discrepancies may result from cross-reactivity and interference in complex biological matrices. Therefore, the present findings should be interpreted with caution and should be validated using gold-standard lipidomic techniques.

The study did not include stratification according to symptom severity (e.g., CCS classification), which may influence inflammatory mediator profiles and their clinical interpretation.

Therefore, the results should be interpreted with caution and validated using more precise analytical techniques. Finally, circulating mediator levels may not fully reflect local vascular or plaque-specific processes, which remain to be elucidated in mechanistic studies.

Although multivariable logistic regression identified independent associations with angiographically confirmed coronary artery disease, the relatively limited number of outcome events compared with the number of candidate variables increases the possibility of model overfitting. Consequently, these regression models should be considered exploratory and hypothesis-generating rather than definitive predictive models. Independent validation in larger cohorts is required.

It is worth highlighting that the present study was conducted in patients with chronic coronary syndrome; the concept of inflammatory–resolution imbalance may extend to other chronic inflammatory disorders. Future studies should determine whether optimizing lifestyle factors known to influence systemic inflammation—including regular physical activity, maintenance of a healthy body weight, smoking cessation, and diets rich in omega-3 polyunsaturated fatty acids—also promotes endogenous resolution pathways. However, the current findings should not be interpreted as evidence supporting specific therapeutic recommendations until prospective interventional studies become available.

## 4. Materials and Methods

### 4.1. Characteristics of the Study Group

The study included 83 consecutive patients hospitalized in the Department of Arterial Hypertension and Internal Medicine due to stable angina or angina-equivalent symptoms. Venous blood samples were collected from all patients upon admission for routine laboratory testing and determination of serum concentrations of selected lipid mediators. Echocardiography and coronary angiography were also performed in all patients. The relationships between the concentrations of the studied mediators and the patients’ clinical, angiographic, and cardiometabolic profiles were then analyzed, with particular attention to differences by sex, age, and body mass index (BMI).

### 4.2. Measurement of Circulating Lipid Mediators

Peripheral venous blood was collected on admission, before coronary angiography and before any study-specific intervention. Blood samples were obtained using standard serum collection tubes and allowed to clot for up to 5 min at room temperature. Samples were subsequently centrifuged at 4000 rpm for 5 min at room temperature. The serum fraction was carefully separated, aliquoted into polypropylene tubes to minimize repeated handling, and stored at −80 °C until analysis. Samples underwent no more than one freeze–thaw cycle(s). Grossly hemolyzed, lipemic, or insufficient samples were excluded from the analytical procedure.

Concentrations of Leukotriene B4 (LTB4), maresin 1 (MaR1), resolvin E1 (RvE1), resolvin D1 (RvD1), and prostaglandin E2 (PGE2) were determined as defined in the enzyme-linked immunosorbent assay (ELISA) kit toolkits published by Wuhan Enlibio Biotech Co., Ltd. (Wuhan, China). The following kits were used: Human LTB4 ELISA Kit, catalog number EIA05989h; Human MaR1 ELISA Kit, catalog number EIA07987h; Human RvE1 ELISA Kit, catalog number EIA06547h; Human RvD1 ELISA Kit, catalog number EIA06548h; and Human PGE2 ELISA Kit, catalog number EIA06212h. All assays were performed according to the manufacturer’s instructions. Before assaying, substances were thawed on ice, gently mixed, and briefly centrifuged to remove insoluble material. Each sample was run in quadruplicate, consisting of three separate units, forming a single unit with an additional set. The applied summary was verified in preliminary studies to confirm that the obtained values covered the analytical range of the calibration curve.

Standards and interpreters were assayed in duplicate, and the arithmetic calculations from two wells were used for further calculations. A blank and a full set of calibration standards can be run on each master plate. A separate calibration curve is generated for each endpoint based on the standards provided by the supplier. The assay ranges were 15.6–1000 pg/mL for LTB4, 3.12–200 ng/mL for MaR1, 0.312–20 ng/mL for RvE1, 31.2–2000 pg/mL for RvD1, and 7.8–500 pg/mL for PGE2. The manufacturer’s declared analytical values, provided by disclosing detectable concentrations, were 5 pg/mL, 0.6 ng/mL, 0.06 ng/mL, 12 pg/mL, and 1 pg/mL, respectively. Optical density was measured at 450 nm within 10 min of accession addition using a calibrated microplate reader. The absorbance value of the standards was subtracted from the blank. Analytical concentrations were determined by interpolation from the critical calibration curve on the same plate using curve-fitting software. Final concentration values were corrected to account for a fourfold increase in performance. Results with a signal below the declared analytical sensitivity switch were reported as outside the limits of detection (<LOD). Interpolated values below the calibration curve point but above the range limit were classified as outside the calibration range limit and were not considered quantitative results. According to the specifications, the intra-assay consistency coefficient is ≤8%, while the inter-assay consistency coefficient is ≤12% for each of the five assays. The supplier’s declared recovery range is 70–110% for all kits. These features are available, provided they are available within the parameters being tested.

Assay performance was monitored based on blank test results and calibration criteria obtained on each plate. Concentrations are calculated solely from the calibration curve on the same plate as the one analyzed. Recovery data and analytical parameters are based on manufacturer documentation. No intra-laboratory analysis of components, scattered conclusions, or controller interference is allowed. Due to limited access to potential pro-resolution mediators and the potential for cross-reactivity and matrix-related interference, ELISA values should be interpreted as an indicator of immunoreactivity in circulation, rather than as a composite of the structural identities of individual lipid mediators. The assays did not provide chromatographic separation or molecular mass confirmation. Accordingly, the analytical method cannot fully exclude cross-reactivity with structurally related lipid metabolites. Liquid chromatography–tandem mass spectrometry remains the reference technique for confirmatory identification and quantitative lipidomic profiling, and future studies should validate the present observations using targeted LC–MS/MS.

### 4.3. Echocardiography and Coronary Angiography

Echocardiography was performed in all patients according to current diagnostic standards. Basic morphological and functional parameters of the heart were assessed. Additionally, subjects underwent coronary angiography to assess the presence and severity of coronary artery lesions and to determine eligibility for further treatment.

### 4.4. Statistical Analysis

Statistical analyses were performed using SPSS software, version 23 (IBM SPSS Statistics, New York, NY, USA). Continuous variables were expressed as medians with interquartile ranges (Q1–Q3). Q1 and Q3 represent the first and third quartiles, respectively.

Comparisons between two independent groups were performed using the Mann–Whitney U test because the distributions were not normally distributed. Categorical variables were compared using the chi-square test or Fisher’s exact test where appropriate. To control for multiple comparisons, *p*-values were adjusted using the Benjamini–Hochberg false discovery rate (FDR) procedure. Adjusted q-values < 0.05 were considered statistically significant.

Multivariable logistic regression models were constructed using a stepwise selection approach. Variables included in the models were selected based on clinical relevance and univariate significance (*p* < 0.10). Odds ratios (ORs) with 95% confidence intervals (CIs) were calculated. Model discrimination was assessed using receiver operating characteristic (ROC) curve analysis and area under the curve (AUC).

### 4.5. Ethical Aspects

The study was conducted in accordance with the principles of the Declaration of Helsinki and after obtaining approval from the Institutional Ethics Committee of Poznan University of Medical Sciences (protocol code 113/21; 6 November 2021). All participants gave informed consent to participate in the study.

## 5. Conclusions

In conclusion, patients with chronic coronary syndrome may exhibit distinct inflammatory–resolution mediator profiles that vary according to age, sex, and obesity status. The simultaneous elevation of LTB4 and specialized pro-resolving mediators suggests activation of compensatory resolution pathways in response to persistent inflammatory stimuli. These findings support a model of altered inflammatory–resolution coupling in CCS rather than simple inflammatory excess. These observations are consistent with altered regulation of inflammatory and pro-resolving pathways but do not establish impaired inflammatory resolution. Rather than representing a final observation, these results provide a conceptual framework for future investigations aimed at understanding how inflammatory resolution becomes dysregulated during chronic vascular disease. Defining the biological mechanisms that restore effective inflammatory resolution may ultimately enable preventive strategies and personalized, resolution-directed therapies that modify disease progression rather than simply control its consequences.

Future studies incorporating healthy controls, disease-severity assessment, and mass spectrometry-based lipidomics are required to determine whether these mediator networks contribute directly to disease progression and clinical outcomes.

## Figures and Tables

**Table 1:** Spearman correlation coefficients (r) and corresponding *p*-values among circulating lipid mediators.

Variable	LTB4	MaR1	RvE1	RvD1	PGE2
LTB4	—	r = 0.761 *p* < 0.001	r = −0.079 *p* = 0.514	r = 0.762 *p* < 0.001	r = −0.019 *p* = 0.879
MaR1	r = 0.761 *p* < 0.001	—	r = −0.094 *p* = 0.407	r = 0.749 *p* = < 0.001	r = −0.080 *p* = 0.486
RvE1	r = −0.079 *p* = 0.514	r = −0.094 *p* = 0.407	—	r = −0.069 *p* = 0.555	r = 0.177 *p* = 0.102
RvD1	r = 0.762 *p* < 0.001	r = 0.749 *p* = < 0.001	r = −0.069 *p* = 0.555	—	r = 0.025 *p* = 0.830
PGE2	r = −0.019 *p* = 0.879	r = −0.080 *p* = 0.486	r = 0.177 *p* = 0.102	r = 0.025 *p* = 0.830	—

Abbreviations: LTB4—leukotriene B4; MaR1—maresin-1; PGE2—prostaglandin E2; RvD1—resolvin D1; RvE1—resolvin E1.

**Table 2:** Clinical and angiographic characteristics of women and men.

Variable	Men (n = 29)	Women (n = 54)	*p*
Age (years) (median (Q1–Q3))	73 (69–77)	67 (63–77)	0.158
Hypertension (n (%))	29 (100.00%)	52 (96.27%)	0.540
Dyslipidemia (n (%))	25 (86.21%)	52 (96.27%)	0.177
Diabetes mellitus (n (%))	5 (17.24%)	3 (5.56%)	0.951
Stroke (n (%))	3 (10.34%)	12 (22.22%)	0.384
Peripheral artery disease (PAD) (n (%))	5 (17.24%)	7 (12.96%)	0.306
Chronic obstructive pulmonary disease (COPD) (n (%))	9 (31.03%)	31 (57.41%)	0.750
Weight (kg) (median (Q1–Q3))	73.00 (69.00–84.00)	83.50 (77.00–93.00)	0.006
Height (cm) (median (Q1–Q3))	160.00 (156.00–166.00)	173.50 (170.00–176.00)	<0.001
BMI (kg/m^2^) (median (Q1–Q3))	30.48 (25.97–32.00)	28.37 (25.35–30.42)	0.080
Smoking (n (%))	2 (6.90%)	10 (18.52%)	0.390

Abbreviations: BMI—body mass index, cm—centimeter, kg—kilogram, m^2^—square meter, Q—quartile.

**Table 3:** Laboratory parameters and lipid mediators in women and men.

Variable	Men (n = 29)	Women (n = 54)	*p*	FDR q
WBC (×10^9^/L) (median (Q1–Q3))	7.17 (5.46–9.21)	7.29 (6.12–8.80)	0.923	0.936
Neutrophils (%) (median (Q1–Q3))	62.40 (55.90–67.00)	61.90 (55.70–69.80)	0.596	0.816
Lymphocytes (%) (median (Q1–Q3))	23.70 (20.90–30.00)	25.20 (19.00–30.70)	0.707	0.865
Monocytes (%) (median (Q1–Q3))	8.10 (7.10–11.00)	9.00 (7.80–10.50)	0.359	0.646
NLR (median (Q1–Q3))	2.71 (1.89–3.20)	2.45 (1.84–4.02)	0.671	0.850
MLR (median (Q1–Q3))	0.33 (0.27–0.38)	0.39 (0.31–0.51)	0.133	0.319
Hemoglobin (mmol/L) (median (Q1–Q3))	8.40 (8.10–9.00)	8.70 (7.30–9.40)	0.847	0.936
Hematocrit (L/L) (median (Q1–Q3))	0.41 (0.39–0.44)	0.41 (0.35–0.45)	0.475	0.722
MCHC (mmol/L) (median (Q1–Q3))	20.70 (20.10–20.80)	20.80 (20.40–21.20)	0.097	0.291
RDW, (%) (median (Q1–Q3))	13.40 (12.80–14.00)	13.20 (12.70–14.10)	0.721	0.865
Platelets (×10^9^/L) (median (Q1–Q3))	216.00 (178.00–289.00)	207.00 (175.00–253.00)	0.266	0.596
MPV (fL) (median (Q1–Q3))	10.60 (10.00–11.20)	10.60 (10.00–11.30)	0.992	0.992
ALT (U/L) (median (Q1–Q3))	26.00 (19.00–37.00)	23.50 (18.00–37.00)	0.868	0.936
Total cholesterol (mg/dL) (median (Q1–Q3))	141.00 (130.00–173.00)	129.50 (116.00–165.00)	0.064	0.256
Glucose (mg/dL) (median (Q1–Q3))	99.00 (87.00–116.00)	99.00 (91.00–117.00)	0.823	0.936
HDL cholesterol (mg/dL) (median (Q1–Q3))	56.00 (43.00–62.00)	48.00 (40.00–60.00)	0.108	0.291
Castelli index (median (Q1–Q3))	3.04 (2.65–3.59)	2.84 (2.52–3.29)	0.228	0.548
Creatinine (mg/dL) (median (Q1–Q3))	0.89 (0.69–1.15)	0.89 (0.74–1.05)	0.861	0.936
Triglycerides (mg/dL) (median (Q1–Q3))	89.00 (67.00–120.00)	105.50 (75.00–126.00)	0.276	0.596
Uric acid (mg/dL) (median (Q1–Q3))	5.20 (4.50–6.10)	5.20 (4.30–6.37)	0.923	0.936
HbA1c (%) (median (Q1–Q3))	6.10 (5.80–7.50)	6.20 (5.60–7.20)	0.813	0.936
LDL cholesterol (mg/dL) (median (Q1–Q3))	76.00 (56.00–103.00)	74.00 (59.00–103.00)	0.936	0.936
LTB4 (pg/mL) (median (Q1–Q3))	320.37 (157.50–654.41)	128.70 (40.18–216.72)	<0.001	<0.001
MaR1 (ng/mL) (median (Q1–Q3))	54.66 (26.56–69.56)	15.67 (6.52–29.32)	<0.001	<0.001
RvE1 (ng/mL) (median (Q1–Q3))	5.49 (4.72–5.75)	6.43 (5.64–9.21)	<0.001	<0.001
RvD1 (pg/mL) (median (Q1–Q3))	646.70 (434.30–798.89)	245.45 (145.50–611.34)	<0.001	<0.001
PGE2 (pg/mL) (median (Q1–Q3))	181.98 (152.77–231.22)	203.25 (175.24–242.26)	0.133	0.319

Abbreviations: ALT—alanine aminotransferase; HbA1c—glycated hemoglobin; dL—deciliter; HDL—high-density lipoprotein cholesterol; LTB-4—leukotriene B4; LDL—low-density lipoprotein cholesterol; mg—milligram; mmol—millimole; L—liter; MaR1—maresin-1; MCHC—mean corpuscular hemoglobin concentration; MLR—monocyte-to-lymphocyte ratio; MPV—mean platelet volume; ng—nanogram; NLR—neutrophil-to-lymphocyte ratio; pg—picogram; PGE-2—prostaglandin E2; RDW—red cell distribution width; RvD-1—resolvin D1; RvE-1—resolvin E1; WBC—white blood cell count.

**Table 4:** Clinical and angiographic characteristics of patients with BMI < 30 and BMI ≥ 30.

Variable	BMI < 30 kg/m^2^ (n = 49)	BMI ≥ 30 kg/m^2^ (n = 34)	*p*
Age (years) (median (Q1–Q3))	71.00 (66.00–74.00)	70.00 (61.00–76.00)	0.487
Sex (M/F) (n/n)	14/35	15/19	0.232
Hypertension (n (%))	48 (97.96%)	33 (97.06%)	1.000
Diabetes mellitus (n (%))	20 (40.82%)	11 (32.35%)	0.516
Stroke (n (%))	4 (8.16%)	4 (11.76%)	0.786
Peripheral artery disease (PAD) (n (%))	11 (22.45%)	5 (14.71%)	0.552
Chronic obstructive pulmonary disease (COPD) (n (%))	6 (12.24%)	6 (17.65%)	0.682
Body weight (kg) (median (Q1–Q3))	76.00 (67.00–82.00)	91.00 (83.00–95.00)	<0.001
Height (cm) (median (Q1–Q3))	172.00 (165.00–176.00)	168.50 (161.00–174.00)	0.128
Coronary angiography confirmed coronary changes (n (%))	24 (49.00%)	10 (29.41%)	0.136
Smoking (n (%))	5 (10.20%)	7 (20.59%)	0.425

Abbreviations: BMI—body mass index, cm—centimeter, kg—kilogram, m^2^—square meter, Q—quartile.

**Table 5:** Laboratory parameters and lipid mediators in the BMI <3 0 and BMI ≥ 30 groups.

Variable	BMI < 30 (n = 49)	BMI ≥ 30 (n = 34)	*P*	q
WBC (×10^9^/L) (median (Q1–Q3))	7.45 (6.19–8.84)	6.85 (5.53–9.14)	0.456	0.741
Neutrophils (%) (median (Q1–Q3))	61.80 (55.60–69.70)	62.40 (55.90–67.00)	0.657	0.870
Lymphocytes (%) (median (Q1–Q3))	25.15 (18.45–31.05)	24.05 (20.20–30.00)	0.623	0.870
Monocytes (%) (median (Q1–Q3))	9.15 (7.75–10.55)	8.10 (7.40–10.50)	0.254	0.559
NLR (median (Q1–Q3))	2.52 (1.81–4.20)	2.64 (1.89–3.30)	0.577	0.870
MLR (median (Q1–Q3))	0.39 (0.31–0.53)	0.33 (0.27–0.41)	0.056	0.205
Hemoglobin (mmol/L) (median (Q1–Q3))	8.70 (7.20–9.45)	8.40 (7.90–9.00)	0.922	0.922
Hematocrit (L/L) (median (Q1–Q3))	0.41 (0.35–0.45)	0.41 (0.39–0.43)	0.855	0.922
MCHC (mmol/L) (median (Q1–Q3))	20.80 (20.40–21.25)	20.65 (20.20–21.00)	0.078	0.215
RDW, (%) (median (Q1–Q3))	13.25 (12.70–14.10)	13.35 (12.80–14.00)	0.797	0.922
Platelets (×10^9^/L) (median (Q1–Q3))	206.00 (163.50–247.50)	235.00 (191.00–282.00)	0.079	0.215
MPV (fL) (median (Q1–Q3))	10.65 (10.10–11.35)	10.55 (10.00–11.20)	0.565	0.870
ALT (U/L) (median (Q1–Q3))	24.00 (20.00–36.00)	25.00 (18.00–37.00)	0.814	0.922
Total cholesterol (mg/dL) (median (Q1–Q3))	129.00 (116.00–155.00)	142.00 (129.00–183.00)	0.016	0.070
Glucose (mg/dL) (median (Q1–Q3))	100.00 (88.00–117.00)	97.50 (90.00–110.00)	0.603	0.870
HDL cholesterol (mg/dL) (median (Q1–Q3))	46.00 (39.00–57.00)	56.50 (46.00–64.00)	0.010	0.044
Castelli index (median (Q1–Q3))	2.84 (2.56–3.29)	2.97 (2.41–3.59)	0.504	0.741
Creatinine (mg/dL) (median (Q1–Q3))	0.90 (0.76–1.05)	0.83 (0.69–1.13)	0.330	0.662
Triglycerides (mg/dL) (median (Q1–Q3))	110.00 (79.00–126.00)	86.50 (63.00–120.00)	0.099	0.233
Uric acid (mg/dL) (median (Q1–Q3))	5.30 (4.20–6.45)	5.10 (4.30–6.10)	0.797	0.922
HbA1c (%) (median (Q1–Q3))	6.30 (5.60–7.20)	6.00 (5.75–7.10)	0.804	0.922
LDL cholesterol (mg/dL) (median (Q1–Q3))	75.00 (59.00–98.00)	75.00 (56.00–104.00)	0.930	0.930
LTB4 (pg/mL) (median (Q1–Q3))	132.08 (40.17–211.37)	303.39 (122.33–654.41)	0.002	0.011
MaR1 (ng/mL) (median (Q1–Q3))	15.67 (6.83–25.06)	54.66 (23.38–74.91)	<0.001	<0.001
RvE1 (ng/mL) (median (Q1–Q3))	6.60 (5.72–9.36)	5.48 (4.74–5.89)	<0.001	<0.001
RvD1 (pg/mL) (median (Q1–Q3))	242.64 (145.50–408.16)	650.54 (391.24–798.89)	<0.001	<0.001
PGE2 (pg/mL) (median (Q1–Q3))	201.49 (173.22–241.10)	192.43 (155.55–236.09)	0.528	0.741

Abbreviations: ALT—alanine aminotransferase; HbA1c—glycated hemoglobin; dL—deciliter; HDL—high-density lipoprotein cholesterol; LTB-4—leukotriene B4; LDL—low-density lipoprotein cholesterol; mg—milligram; mmol—millimole; L—liter; MaR1—maresin-1; MCHC—mean corpuscular hemoglobin concentration; MLR—monocyte-to-lymphocyte ratio; MPV—mean platelet volume; ng—nanogram; NLR—neutrophil-to-lymphocyte ratio; pg—picogram; PGE-2—prostaglandin E2; RDW—red cell distribution width; RvD-1—resolvin D1; RvE-1—resolvin E1; WBC—white blood cell count.

**Table 6:** Clinical and angiographic characteristics of patients <65 and ≥65 years of age.

Variable	<65 Years (n = 24)	≥65 Years (n = 59)	*p*
Age (years) (median (Q1–Q3))	59.50 (55.50–62.50)	73.00 (70.00–77.00)	<0.001
Sex (F/M) (n/n)	7/17	22/37	0.566
Hypertension (n (%))	22 (91.67%)	55 (93.22%)	0.520
Diabetes mellitus (n (%))	8 (33.33%)	23 (38.98%)	0.694
Stroke (n (%))	0 (0.00%)	8 (13.56%)	0.340
Peripheral artery disease (PAD) (n (%))	5 (20.83%)	11 (18.64%)	0.877
Chronic obstructive pulmonary disease (COPD) (n (%))	3 (12.50%)	9 (15.25%)	0.846
Body weight (kg) (median (Q1–Q3))	88.50 (79.00–97.00)	80.00 (70.00–88.00)	0.010
Height (cm) (median (Q1–Q3))	172.50 (168.00–176.00)	170.00 (162.00–175.00)	0.129
BMI (kg/m^2^) (median (Q1–Q3))	30.25 (26.47–32.31)	28.37 (25.08–31.11)	0.074
Coronary angiography confirmed coronary changes (n (%))	4 (16.67%)	30 (50.85%)	<0.001
Smoking (n (%))	7 (29.17%)	5 (8.47%)	0.142

Abbreviations: BMI—body mass index, cm—centimeter, kg—kilogram, m^2^—square meter, Q—quartile.

**Table 7:** Laboratory parameters and lipid mediators in patients <65 and ≥65 years of age.

Variable	Age < 65 Years (n = 24)	Age ≥ 65 Years (n = 59)	*P*	FDR q
WBC (×10^9^/L) (median (Q1–Q3))	6.77 (6.12–9.26)	7.46 (5.63–8.82)	0.765	0.907
Neutrophils (%) (median (Q1–Q3))	61.80 (55.10–68.70)	62.40 (56.70–67.90)	0.859	0.907
Lymphocytes (%) (median (Q1–Q3))	25.15 (20.20–30.65)	24.05 (19.00–31.30)	0.996	0.996
Monocytes (%) (median (Q1–Q3))	9.45 (7.75–10.55)	8.65 (7.70–10.50)	0.338	0.676
NLR (median (Q1–Q3))	2.62 (1.80–3.94)	2.51 (1.89–3.71)	0.820	0.907
MLR (median (Q1–Q3))	0.36 (0.31–0.48)	0.36 (0.28–0.52)	0.697	0.907
Hemoglobin (mmol/L) (median (Q1–Q3))	8.75 (7.20–9.55)	8.55 (7.80–9.10)	0.980	0.996
Hematocrit (L/L) (median (Q1–Q3))	0.41 (0.34–0.46)	0.41 (0.38–0.44)	0.828	0.907
MCHC (mmol/L) (median (Q1–Q3))	21.10 (20.65–21.30)	20.70 (20.30–21.00)	0.011	0.048
RDW, (%) (median (Q1–Q3))	13.20 (12.75–13.70)	13.40 (12.80–14.20)	0.294	0.662
Platelets (×10^9^/L) (median (Q1–Q3))	213.00 (163.50–247.50)	212.00 (180.00–280.00)	0.381	0.705
MPV (fL) (median (Q1–Q3))	10.50 (10.10–11.25)	10.60 (10.00–11.20)	0.915	0.996
ALT (U/L) (median (Q1–Q3))	22.50 (15.00–36.50)	26.00 (19.00–37.00)	0.310	0.662
Total cholesterol (mg/dL) (median (Q1–Q3))	123.00 (109.00–133.00)	141.00 (125.00–180.00)	<0.001	<0.001
Glucose (mg/dL) (median (Q1–Q3))	98.50 (86.50–118.50)	99.00 (92.00–116.00)	0.776	0.907
HDL cholesterol (mg/dL) (median (Q1–Q3))	41.50 (36.50–46.00)	56.00 (46.00–63.00)	<0.001	<0.001
Castelli index (median (Q1–Q3))	2.82 (2.57–3.29)	2.88 (2.38–3.45)	0.964	0.996
Creatinine (mg/dL) (median (Q1–Q3))	0.96 (0.81–1.17)	0.84 (0.69–1.12)	0.027	0.087
Triglycerides (mg/dL) (median (Q1–Q3))	89.00 (74.50–135.50)	105.00 (78.00–121.00)	0.738	0.907
Uric acid (mg/dL) (median (Q1–Q3))	5.50 (3.90–6.60)	5.20 (4.30–6.20)	0.689	0.907
HbA1c (%) (median (Q1–Q3))	6.10 (5.60–7.60)	6.20 (5.70–6.80)	0.874	0.907
LDL cholesterol (mg/dL) (median (Q1–Q3))	71.00 (55.50–88.50)	81.00 (58.00–109.00)	0.377	0.705
LTB4 (pg/mL) (median (Q1–Q3))	92.65 (26.54–193.36)	217.95 (94.57–532.86)	0.007	0.035
MaR1 (ng/mL) (median (Q1–Q3))	11.05 (4.73–21.26)	34.73 (14.75–64.62)	<0.001	<0.001
RvE1 (ng/mL) (median (Q1–Q3))	8.59 (5.95–11.03)	5.69 (4.90–6.50)	<0.001	<0.001
RvD1 (pg/mL) (median (Q1–Q3))	221.37 (123.99–363.32)	475.80 (260.82–780.45)	0.011	0.048
PGE2 (pg/mL) (median (Q1–Q3))	202.44 (181.22–228.50)	194.49 (155.55–243.75)	0.694	0.907

Abbreviations: ALT—alanine aminotransferase; HbA1c—glycated hemoglobin; dL—deciliter; HDL—high-density lipoprotein cholesterol; LTB-4—leukotriene B4; LDL—low-density lipoprotein cholesterol; mg—milligram; mmol—millimole; L—liter; MaR1—maresin-1; MCHC—mean corpuscular hemoglobin concentration; MLR—monocyte-to-lymphocyte ratio; MPV—mean platelet volume; ng—nanogram; NLR—neutrophil-to-lymphocyte ratio; pg—picogram; PGE-2—prostaglandin E2; RDW—red cell distribution width; RvD-1—resolvin D1; RvE-1—resolvin E1; WBC—white blood cell count.

## Data Availability

The database used to generate the study results is available upon reasonable request from the corresponding authors. The restriction is based on ethical limitations and the privacy policy.
